# High-Performance Electrochromic Energy Storage Devices Based on Hexagonal WO_3_ and SnO_2_/PB Composite Films

**DOI:** 10.3390/ma18122871

**Published:** 2025-06-17

**Authors:** Yi Wang, Zilong Zhang, Ze Wang, Yujie Yan, Tong Feng, An Xie

**Affiliations:** 1 Key Laboratory of Functional Materials and Applications of Fujian Province, School of Materials Science and Engineering, Xiamen University of Technology, Xiamen 361024, China; yiwang@xmut.edu.cn (Y.W.); anxie@xmut.edu.cn (A.X.); 2 School of Mechanical Electrical and Information Engineering, Xiamen Institute of Technology, Xiamen 361021, China

**Keywords:** electrochromic, tungsten oxide, hydrothermal, composite films

## Abstract

Electrochromic devices have garnered significant interest owing to their promising applications in smart multifunctional electrochromic energy storage systems (EESDs) and their emerging next-generation electronic technologies. Tungsten oxide (WO_3_), possessing both electrochromic and pseudocapacitive characteristics, offers great potential for developing multifunctional devices with enhanced performance. However, achieving an efficient and straightforward synthesis of WO_3_ electrochromic films, while simultaneously ensuring high coloration efficiency and energy storage capability, remains a significant challenge. In this work, a low-temperature hydrothermal approach is employed to directly grow hexagonal-phase WO_3_ films on FTO substrates. This process utilizes sorbitol to promote nucleation and rubidium sulfate to regulate crystal growth, enabling a one-step in situ fabrication strategy. To complement the high-performance WO_3_ cathode, a composite PB/SnO_2_ film was designed as the anode, offering improved electrochromic properties and enhanced stability. The assembled EESD exhibited fast bleaching/coloration response and a high coloration efficiency of 101.2 cm^2^ C^−1^. Furthermore, it exhibited a clear and reversible change in optical properties, shifting from a transparent state to a deep blue color, with a transmittance modulation reaching 81.47%.

## 1. Introduction

Electrochromism refers to the reversible alteration of a material’s optical properties—such as color, transmittance, and reflectance—induced by an applied electric field [1]. Owing to their reversible and controllable optical properties, electrochromic materials are widely studied for potential uses in smart glazing, responsive screens, military concealment, and automotive rearview mirrors with anti-glare functionality [2]. In recent years, electrochromic energy storage devices (EESDs) have attracted significant research attention due to their unique ability to visually display energy storage status in real time, effectively integrating energy storage with intuitive state monitoring [3]. Notably, conventional electrochromic devices (ECDs) and energy storage systems, such as supercapacitors and batteries, share similarities in device architecture, reaction kinetics, and material selection. These intrinsic correlations have prompted growing interest in the development of multifunctional devices that seamlessly integrate electrochromic and energy storage capabilities, enabling advanced applications in next-generation energy and display technologies [4,5].

Electrochromic materials can be broadly divided into three main types: organic compounds, inorganic materials, and organic–inorganic hybrid systems [6,7]. Among various inorganic materials, WO_3_ distinguishes itself by offering superior coloration performance, a wide range of optical tunability, and strong integration potential with electrochromic and energy storage devices [8]. WO_3_ thin films can be fabricated using multiple approaches, including but not limited to sputtering, electrochemical deposition, template-assisted methods, thermal evaporation, CVD, and hydrothermal synthesis [9,10,11,12,13,14]. Hydrothermal synthesis stands out as a straightforward and effective approach for preparing crystalline oxides characterized by high surface areas and versatile morphologies [15]. This technique provides several advantages, such as tunable particle size, and cost-effectiveness [16]. However, the lattice mismatch between WO_3_ and the underlying substrate often impedes high-quality film formation [17]. Previous research indicates that the hydrothermal growth of WO_3_ films on FTO substrates generally relies on a pre-deposited seed layer—such as a WO_3_ nucleation film or another metal oxide—which adds to the overall complexity and energy demand of the fabrication process [18]. More recently, specific organic additives, such as ethylene glycol and glycerol, have been used to bridge the WO_3_ nuclei and the substrate interface, enabling self-seeding hydrothermal synthesis [19]. Compared to methods requiring pre-deposited seed layers, the direct one-step growth of WO_3_ nanostructures on FTO substrates offers improved charge transport, as the nanostructured layer forms intimate contact with the conductive substrate. The polymorphism of WO_3_ arises from diverse structural configurations of the [WO_6_] octahedra, which typically connect by sharing corners and edges [20]. By assembling these octahedra into distinct lattice architectures, multiple interstitial positions can be created within the crystal structure. Such open structures enable the diffusion of guest ions into the bulk phase, allowing for both surface adsorption/desorption and bulk intercalation/deintercalation processes. Previous studies have demonstrated that the crystal structure of WO_3_ plays a pivotal role in determining its pseudocapacitive behavior and electrochemical performance [21]. Therefore, rational design of the crystal architecture of WO_3_ films is essential for advancing high-performance electrochromic energy storage devices (EESDs). Nevertheless, systematic investigations that integrate one-step fabrication strategies, crystal structure modulation, and interfacial coupling mechanisms for high-efficiency EESD development remain scarce. In our recent work, we introduced an efficient one-step hydrothermal method to directly grow WO_3_ electrochromic films on FTO glass, resulting in excellent performance [22]. However, in that study, the EESD performance was constrained by the limited cycling stability arising from the mismatch in electrochromic behavior between the WO_3_ and PB electrodes. Therefore, while optimizing the WO_3_ cathode remains essential, simultaneously enhancing the performance of the anode is critical to improving both the electrochromic and charge–discharge characteristics of the device.

In typical EESDs, WO_3_ serves as the cathodic electrochromic layer (negative electrode), while a complementary material—such as Prussian blue (PB), polyaniline (PANI), or MnO_2_—is employed as the anodic electrochromic layer (positive electrode) to enhance the overall performance of the device [23]. Among these, PB is particularly attractive due to its complementary color transition with that of WO_3_, switching between a transparent and deep blue state, making it one of the most widely used counterparts in WO_3_-based electrochromic devices [24]. However, PB films fabricated by electrodeposition often suffer from poor cycling stability, primarily due to the dense packing of PB nanoparticles [25]. Constructing composite architectures has proven to be an effective strategy for enhancing the stability and performance of PB films by leveraging the synergistic advantages of multiple components. In this study, we propose the design of a PB/SnO_2_ composite nanostructure as a promising anodic electrochromic material. The porous nanosheet morphology and high specific surface area of SnO_2_ can mitigate nanoparticle aggregation, improve ion accessibility, and enhance the adhesion of PB to the substrate.

Herein, we designed and assembled an electrochromic energy storage device with outstanding performance characteristics. A facile hydrothermal synthesis route was developed to grow hexagonal-phase WO_3_ films directly on FTO substrates in one step, employing sorbitol (SLC) as a self-seeding polyhydroxy molecule and Rb_2_SO_4_ as the capping agent. As the anodic electrode, a SnO_2_/PB composite film was constructed by first synthesizing nanosheet-structured SnO_2_ films via hydrothermal treatment, followed by the electrodeposition of PB onto the SnO_2_ scaffold. The porous nanosheet architecture of SnO_2_ provided abundant redox-active sites and ion diffusion pathways for the subsequently deposited PB nanoparticles. The fabricated EESD showed fast and reversible color changes between clear and deep blue states, featuring swift response, high coloration efficiency, and remarkable stability over multiple cycles. Notably, the device achieved a high coloration efficiency of 101.2 cm^2^ C^−1^ and an areal capacitance of 4.98 mF cm^−2^ at a current density of 0.05 mA cm^−2^, demonstrating outstanding electrochromic and energy storage performance.

## 2. Results and Discussion

### 2.1. Fabrication and Characterization of WO_3_ Cathodic Films

**Figure 1:**
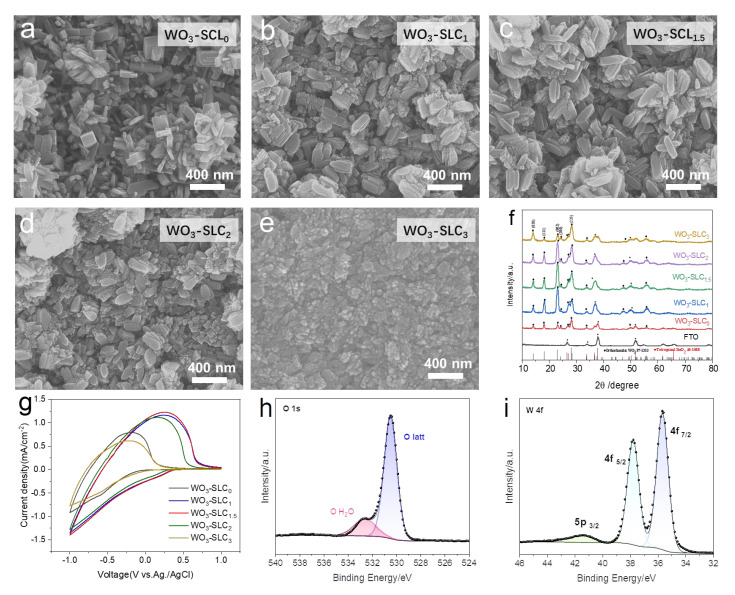
(**a**–**e**) SEM images of WO_3_ films synthesized with different self-seeding agent ratios: (**a**) WO_3_-SLC_0_, (**b**) WO_3_-SLC_1_, (**c**) WO_3_-SLC_1.5_, (**d**) WO_3_-SLC_2_, and (**e**) WO_3_-SLC_3_. (**f**) XRD patterns of the WO_3_ films on FTO substrates with varying SLC ratios. (**g**) Cyclic voltammetry (CV) curves of the WO_3_ electrodes. (**h**) High-resolution XPS spectrum of O 1s. (**i**) High-resolution XPS spectrum of W 4f.

To enable seed-layer-free fabrication via a one-step hydrothermal method, the organic molecule SLC was introduced as a self-seeding agent to direct the in situ growth of WO_3_ thin films. Organic and inorganic additives are known to significantly influence the morphology and crystallization behavior of WO_3_ [26]. To investigate the effect of SLC content, we systematically prepared a series of WO_3_ films from precursor solutions containing different sorbitol loadings and examined their surface morphologies via scanning electron microscopy (SEM), as shown in Figure 1a–e. The samples were labeled WO_3_-SLC_0_, WO_3_-SLC_1_, WO_3_-SLC_1.5_, WO_3_-SLC_2_, and WO_3_-SLC_3_, corresponding to 0 g, 1 g, 1.5 g, 2 g, and 3 g of sorbitol added to the precursor solution, respectively. Details of the synthesis procedure are provided in the Experimental section. As illustrated in Figure 1a, the WO_3_ film synthesized without SLC exhibits a disordered array of rectangular nanobricks, which is typical for hydrothermally grown WO_3_ in the absence of any nucleation-promoting additives [16]. Upon increasing the SLC content, the WO_3_ film morphology underwent pronounced changes (Figure 1b–e). Specifically, the sharp edges of the rectangular nanobricks became smoother, and their thickness was gradually reduced. When the SLC amount reached 3 g, the morphology was transformed into densely packed, fine microparticles (Figure 1e), indicating a significant morphological transition induced by high concentrations of SLC.

The crystalline phases of WO_3_ thin films prepared with different concentrations of SLC were further analyzed by X-ray diffraction (XRD), as illustrated in Figure 1f. All samples exhibit characteristic diffraction peaks corresponding to orthorhombic WO_3_ (JCPDS No. 87-1203). Peaks marked with “•” originate from SnO_2_ (JCPDS No. 46-1088), stemming from the underlying FTO substrate. The orthorhombic WO_3_ peaks, indicated by “◆”, are observed at 2θ = 24.2° and 28.1°, corresponding to the (200) and (220) lattice planes, respectively [20]. Compared with the WO_3_-SLC_0_ sample, the addition of SLC does not alter the overall crystal phase of WO_3_. However, variations in the relative peak intensities were observed with increasing SLC content. Specifically, the diffraction intensities of the (111) and (020) planes decrease, while that of the (002) plane shows a notable enhancement. These results indicate that while SLC does not alter the crystalline phase of WO_3_, it significantly modifies the film’s morphology and preferred crystal orientation.

The electrochemical performance of WO_3_ films with different SLC contents was investigated via cyclic voltammetry (CV) in a three-electrode system using 1 M LiClO_4_/PC as the electrolyte, a platinum foil as the counter electrode, and Ag/AgCl as the reference electrode. As depicted in Figure 1g, the representative CV curves of the WO_3_-based electrodes recorded at 50 mV s^−1^ exhibit prominent redox peaks, which correspond to the reversible Faradaic reactions involving electrolyte ion intercalation and deintercalation in the WO_3_ films [27]. With the addition of SLC, the enclosed area of the CV curves increases, indicating enhanced Li^+^ ion insertion and extraction. Among the samples, the WO_3_-SLC_1.5_ film exhibits the largest enclosed area. The variation in the enclosed area can be attributed, in part, to differences in the loading amount of active material on the substrate. Figure S1 displays cross-sectional SEM images of the WO_3_-SLC_x_ films, and the thickness measurements are compiled in Table S1. Under identical fabrication conditions, the WO_3_-SLC_1.5_ sample, which exhibited the highest charge storage capacity, was selected for further testing and analysis. The surface chemical composition and oxidation states of the as-prepared WO_3_-SLC_1.5_ film were characterized by X-ray photoelectron spectroscopy (XPS). As shown in the survey spectrum (Figure S2), the signals corresponding to W, O, and adventitious carbon—commonly introduced from environmental exposure—are clearly observed. The oxygen-related peaks at 530.7 eV and 532.32 eV in Figure 1h are attributed to the lattice oxygen in WO_3_, hydroxyl groups, and surface oxygen species. Figure 1i shows the W 4f XPS spectrum, with characteristic peaks at 35.9 eV, 38.02 eV, and 41.59 eV, assigned to W 4f_7_/_2_, W 4f_5_/_2_, and W 5p_3_/_2_, confirming the W^6^^+^ oxidation state [28].

**Figure 2:**
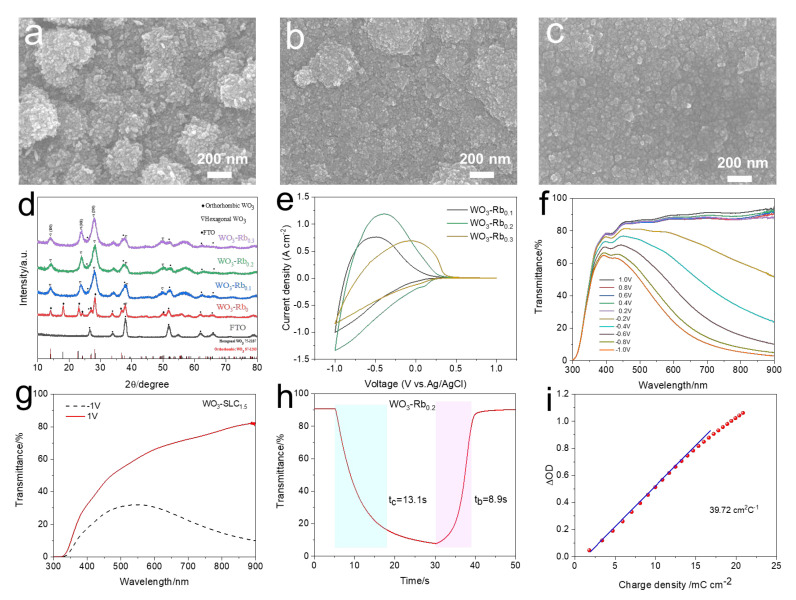
(**a**–**c**) SEM images of WO_3_-Rb_x_ films synthesized with different Ru_2_SO_4_ doping concentrations: (**a**) WO_3_-Rb_0.1_, (**b**) WO_3_-Rb_0.2_, and (**c**) WO_3_-Rb_0.3_. (**d**) XRD patterns of WO_3_-Rb_x_ films on FTO substrates. (**e**) CV curves of the WO_3_-Rb_x_ electrodes. (**f**) In situ transmittance spectra of WO_3_-Rb_0.2_ electrode at various applied voltages from 1.0 V to –1.0 V. (**g**) UV–Vis transmittance spectra of WO_3_-SLC_1.5_ under –1 V (colored) and 1 V (bleached) states. (**h**) Response time measured for the WO_3_-Rb_0.2_ film. (**i**) Variation in the optical density (ΔOD) vs charge density of WO_3_-Rb_0.2_ film.

The electrochemical performance of WO_3_ is typically correlated with its crystal structure. Hexagonal-phase crystalline WO_3_, with its open framework, exhibits superior ion intercalation kinetics and capacity, as evidenced by its higher Li^+^ diffusion coefficient [16]. Regarding crystal composition, different polymorphs of WO_3_ are formed through the corner- and edge-sharing of the [WO_6_] octahedra, which in turn influence their electrochemical properties. In addition, Rb_2_SO_4_ was incorporated into the precursor solution—based on the synthesis conditions of WO_3_-SLC_1.5_—to serve as a capping agent for modulating the crystal growth of the WO_3_ films. Figure 2a–c illustrates the surface morphology variations in the films as a function of the Rb_2_SO_4_ content. Upon the addition of Rb_2_SO_4_, the WO_3_ films exhibited a morphological shift from vertically aligned nanosheets to aggregated nanoparticles. As the amount of Rb_2_SO_4_ increased, the nanoparticles became finer and more uniformly distributed, producing a smoother film surface. The enhanced surface area arising from the smaller particles offered additional electrochemical reaction sites, thereby boosting the film’s electrochemical activity. Relevant cross-sectional SEM micrographs are shown in Figure S3, with thickness data summarized in Table S2. With increasing SLC content, the film thickness gradually increases. XRD analysis results of WO_3_ films synthesized under different Rb_2_SO_4_ conditions are shown in Figure 2d. While the overall diffraction profiles of hexagonal and orthorhombic WO_3_ are largely similar, a sharp peak at 18.1° serves as a fingerprint for the (111) reflection of orthorhombic WO_3_, enabling phase distinction. According to the XRD data in Figure 2d, the sample prepared without Rb_2_SO_4_ (WO_3_-SLC_0_) displays diffraction features characteristic of orthorhombic hydrated WO_3_ (JCPDS No. 87-1203), marked by a distinct peak at 18.1°. As the SLC content increases, a gradual phase transition to hexagonal WO_3_ is observed (JCPDS No. 75-2187). The addition of Rb_2_SO_4_ into the precursor likely regulates the crystallization behavior by preferentially binding to specific facets, thereby guiding directional growth and affecting particle aggregation. During film growth, crystal nuclei typically grow preferentially along certain directions to cover high-energy surfaces, thereby reducing the overall surface energy. The ions dissociated from Rb_2_SO_4_ can preferentially adsorb onto the crystal facets parallel to the c-axis of the WO_3_ nuclei, thereby inhibiting growth along these facets. As a result, the facets perpendicular to the c-axis grow more rapidly, promoting preferential growth along the c-axis and leading to the formation of hexagonal-phase WO_3_ films.

Cyclic voltammetry curves of WO_3_-Rb_x_ films are shown in Figure 2e, recorded using a three-electrode setup in 1 M LiClO_4_ dissolved in propylene carbonate (PC). The applied voltage range was from –1 V to +1 V, with a scanning speed of 50 mV/s. In comparison, the WO_3_-Rb_0.2_ electrode exhibits more pronounced redox peaks and a larger enclosed CV area, indicating that the WO_3_-Rb_0.2_ film possesses higher electrochemical activity. To evaluate the electrochromic behavior, in situ optical transmission spectra of the WO_3_-Rb_0.2_ electrode were collected under applied voltages between –1.0 V and 1.0 V, revealing dynamic modulation across the 400–900 nm range (Figure 2f). At 720 nm, the optical transmittance of the film reaches 82.3%. The electrochromic coloring and bleaching behavior of the film without Rb_2_SO_4_ addition was also evaluated, as shown in Figure 2g. At the same wavelength of 720 nm, its optical transmittance reaches only 52.62%, which is significantly lower than that of the film electrodes prepared with Rb_2_SO_4_. In situ dynamic transmittance measurements at 720 nm were employed to evaluate the response speed and cycling reversibility of the WO_3_-Rb_0.2_ film (Figure 2h). A voltage of –1.0 V was used for coloration and +1.0 V for bleaching, each maintained for 25 s. The measured coloration and bleaching times were 13.1 s and 8.9 s, respectively. The coloration efficiency (CE) is defined as the change in optical density (ΔOD) per unit charge density (Q/A) injected into the electrochromic film and can be calculated using the following equations [29]:

CE = ΔOD/(Q/A)(1)

ΔOD = log(T_b_/T_c_)

where CE (cm^2^·C^−1^) is the coloration efficiency, ΔOD is the change in optical density, Q (C) is the charge inserted or extracted per unit area during coloration or bleaching, A (cm^2^) is the active area of the electrochromic film, T_b_ is the transmittance in the bleached state, and T_c_ is the transmittance in the colored state. The CE value can be determined from the slope of the linear fit shown in Figure 2i. The WO_3_-Rb_0.2_ film exhibits a high coloration efficiency of 39.72 cm^2^ C^−1^. In summary, the as-prepared WO_3_-Rb_0.2_ film demonstrates excellent electrochromic performance and holds great promise for applications in energy storage and electrochromic smart windows.

### 2.2. Fabrication and Characterization of SnO_2_/PB Anodic Films

**Figure 3:**
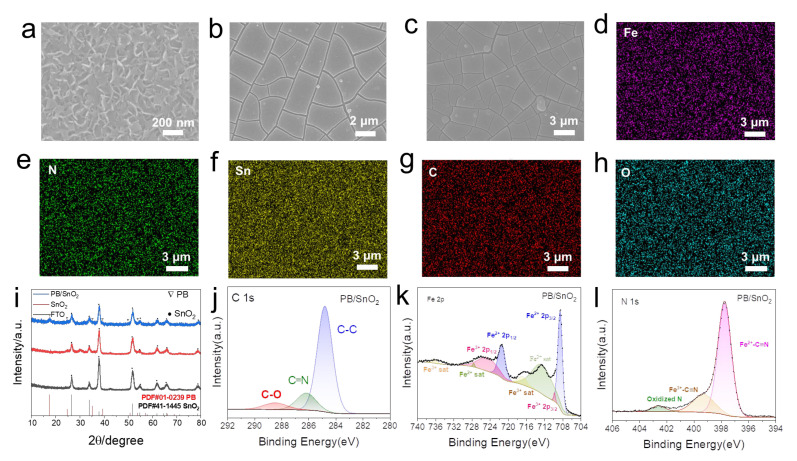
SEM images of (**a**) SnO_2_, (**b**) PB, and (**c**) PB/SnO_2_ composite films, illustrating surface morphology. Energy-dispersive X-ray spectroscopy (EDS) elemental mapping of PB/SnO_2_ film for (**d**) Fe, (**e**) N, (**f**) Sn, (**g**) C, and (**h**) O. (**i**) X-ray diffraction (XRD) patterns of PB/SnO_2_, SnO_2_, and FTO substrates. (**j**–**l**) High-resolution XPS spectrum of C 1s, Fe 2p, and N 1s for PB/SnO_2_.

Prussian Blue (PB) is a well-known anodic electrochromic material with excellent optical modulation properties, and it is commonly paired with WO_3_ cathodic films to construct complementary electrochromic devices. However, PB films are typically fabricated via electrochemical deposition, which often leads to poor cycling stability of the resulting electrodes and limits the overall performance of the devices. Constructing composite thin film electrodes has emerged as an effective strategy to enhance the electrochemical stability of PB-based films. To fabricate PB-based film electrodes with enhanced stability, SnO_2_ films were first synthesized as the substrate layer via a hydrothermal method. The morphology of the as-prepared SnO_2_ films is shown in Figure 3a. The SnO_2_ electrode films possess a nanoflake morphology that offers a high specific surface area, thereby supplying numerous nucleation sites for the subsequent growth of PB. As depicted in Figure 3b, PB was directly electrodeposited onto the FTO substrate. The film exhibits a characteristic cracked, dry-riverbed-like morphology typical of PB films prepared by conventional electrochemical deposition. Using the hydrothermally prepared SnO_2_ film electrode as the substrate, a PB layer was electrodeposited onto its surface via an electrochemical deposition process to form a composite SnO_2_/PB thin film electrode. As shown in Figure 3c, the surface morphology of the composite film exhibits a structure similar to that of the PB film. Further energy-dispersive X-ray spectroscopy (EDS) analysis of the PB/SnO_2_ composite film surface reveals a uniform distribution of Fe, C, N, Sn, and O elements (Figure 3d–h), indicating that the PB layer adheres well to the SnO_2_ nanosheets. Compared to the smooth surface of ITO film electrodes, the large specific surface area of the SnO_2_ nanoflake film electrode significantly enhances the adhesion of PB to the electrode surface, thereby improving its electrochemical performance. The cross-sectional morphologies of the prepared SnO_2_ and SnO_2_/PB films were characterized, as shown in Figure S4, and the corresponding thickness values are summarized in Table S3. 

The crystalline structures of the PB and SnO_2_/PB samples were characterized and confirmed by XRD, as shown in Figure 3i. The characteristic diffraction peaks of PB appear at 2θ = 17.4° and 39.5°, indicating good crystallinity of the PB phase [30]. X-ray photoelectron spectroscopy (XPS) was employed to investigate the surface chemical composition of the SnO_2_/PB sample. As shown in the survey spectrum (Figure S5), elements including Fe, C, N, and O were detected, along with Sn derived from the SnO_2_ nanosheet substrate. Figure 3j displays the deconvoluted C 1s spectrum with peaks at 284.8 eV, 286.2 eV, and 288.5 eV, which correspond to C-C, C≡N, and C-O bonds, respectively. In Figure 3k, the Fe 2p spectrum can be deconvoluted into two pairs of Fe 2p_3_/_2_ and Fe 2p_1_/_2_ peaks, along with their satellite peaks. The Fe 2p spectrum shows Fe^2^^+^ peaks at 708.5 eV (2p_3_/_2_) and 721.4 eV (2p_1_/_2_), while the Fe^3^^+^ peaks appear at 709.8 eV (2p_3_/_2_) and 725.1 eV (2p_1_/_2_) [31]. The N 1s spectrum in Figure 3l exhibits three fitted peaks at 397.8 eV, 399.3 eV, and 402.6 eV, which can be attributed to Fe^2^^+^-C≡N, Fe^3^^+^-C≡N, and N-O bonding, respectively [32].

**Figure 4:**
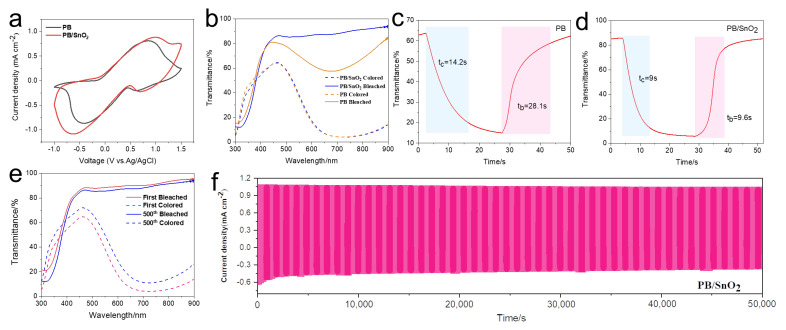
(**a**) CV curves of PB and PB/SnO_2_ films; (**b**) optical transmittance spectra of PB and PB/SnO_2_ films in both colored and bleached states. (**c**,**d**) In situ optical transmittance variation of (**c**) PB and (**d**) PB/SnO_2_ films. (**e**) Transmittance spectra of PB/SnO_2_ films during the first and 500th electrochromic cycles in colored and bleached states. (**f**) Chronoamperometric response of PB/SnO_2_ film.

A direct method to verify the superior performance of the composite film consists of comparing the electrochemical properties of the PB and SnO_2_/PB films. As shown in Figure 4a, the SnO_2_/PB composite film demonstrates a significantly larger CV enclosed area and higher peak currents than those of the PB film, indicating enhanced redox activity and increased charge transfer capacity. The transmittance spectra of the fabricated PB film and PB/SnO_2_ composite film in both their colored and bleached states were measured, as shown in Figure 4b. At 700 nm, the PB film exhibited a colored-state transmittance of 4.31% and a bleached-state transmittance of 58%, resulting in a modulation amplitude of 53.69%. In contrast, the PB/SnO_2_ composite film demonstrated improved optical transmittance and modulation, with a colored-state transmittance of 4.27%, a bleached-state transmittance of 88.08%, and a modulation amplitude of 83.81% at 700 nm—an enhancement of approximately 56% compared to the results for the PB film. Further tests and characterizations were conducted to evaluate the response times of the films, as shown in Figure 4c–d. The PB film exhibited coloring and bleaching times of 14.2 s and 28.1 s, respectively, while the composite PB/SnO_2_ film demonstrated improved response speeds, with coloring and bleaching times of 9.0 s and 9.6 s, respectively. The improved response speed and optical modulation performance indicate that SnO_2_ effectively increases the contact area between PB nanoparticles and the electrolyte, providing more active sites and additional pathways for ion insertion and extraction. The transmittance of the films was tested after a certain number of cycling cycles. As shown in Figure 4e, after 500 cycles, the films exhibited only a slight decrease in transmittance. This indicates good electrochemical cycling stability of the composite film, which can be attributed to the strong interfacial bonding between the SnO_2_ nanosheets and the FTO substrate. Strong adhesion of the PB layer to the SnO_2_ nanosheets prevents particle aggregation and loss, and these elements work in tandem to bolster the electrochemical durability of the composite.

### 2.3. Assembly and Performance Characterization of Electrochromic Devices

**Figure 5:**
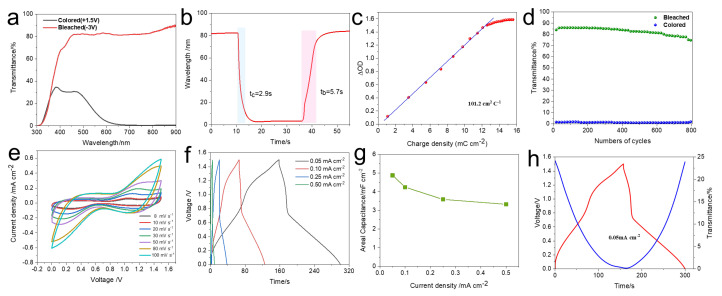
Electrochemical characteristics of the EESD: (**a**) optical transmittance spectra recorded under varying applied voltages; (**b**) time-resolved optical response curves showing the coloration and bleaching dynamics; (**c**) correlation between optical density change (ΔOD) and charge density; (**d**) long-term electrochromic cycling stability during repeated coloration/bleaching; (**e**) cyclic voltammetry curves at different scan rates; (**f**–**g**) galvanostatic charge–discharge (GCD) curves and corresponding areal capacitances at various current densities; (**h**) in-situ transmittance variations synchronized with GCD cycles at a current density of 0.05 mA cm^−2^.

A high-performance EESD was assembled using hydrothermally synthesized WO_3_ and electrodeposited PB/SnO_2_ films, which exhibit complementary optical transitions and matched capacitance. The device reached an optical modulation depth of 81.47% at 720 nm when driven between –3 V and +1.5 V, as presented in Figure 5a. The EESD device exhibits response times of 2.9 s (coloration) and 5.7 s (bleaching) at 720 nm when subjected to +1.3 V and –2.3 V voltage pulses (Figure 5b). The coloration efficiency of the EESD was determined to be 101.2 cm^2^ C^−1^, reflecting its outstanding energy-to-optical conversion capability and suitability for next-generation smart display-integrated storage technologies (Figure 5c). Additionally, the optical transmittance of the EESD was monitored over multiple cycles under alternating –2.3 V/+1.3 V. The device exhibited an initial optical modulation amplitude of 88.06%. After 800 constant-voltage cycles, the modulation amplitude decreased to 74.23%, corresponding to an 84.3% retention of the initial value, demonstrating excellent stability (Figure 5d). To better assess the performance of the fabricated device, a comparison with previously reported electrochromic devices is presented in Figure S5. The results indicate that the device exhibits competitive electrochromic properties relative to those noted in the literature.

In order to explore the energy storage characteristics of the developed EESD while avoiding electrolyte breakdown and polarization, a stable operating window of 0–1.5 V was selected. Figure 5e shows the CV responses recorded over a range of scan rates from 8 to 100 mV s^−1^. Notably, the shapes of the CV profiles remain nearly unchanged, even at the highest scan rates, indicating the excellent rate capability of the EESD. To assess the rate performance of the device, GCD measurements were conducted under various current densities (Figure 5f). The calculated areal capacitances (C_a_) at 0.05, 0.10, 0.25, and 0.50 mA cm^−2^ were 4.98, 4.24, 3.58, and 3.38 mF cm^−2^, respectively, as depicted in Figure 5g. The integration of energy storage and electrochromic functionalities within a single device to serve as an energy-storage indicator is highly attractive, as the device’s color change enables a direct visual estimation of the stored energy level. Figure 5h presents the galvanostatic charge–discharge profile at 0.05 mA cm^−2^, alongside the in situ transmittance, illustrating this compelling and practical concept. During charging, the EESD’s transmittance gradually decreases, whereas it rises during discharging, revealing a clear, reversible optical change that directly correlates with its charge storage state. The EESD’s excellent energy storage and optical switching performance suggests broad potential in multifunctional electronics and display applications.

## 3. Conclusions

In conclusion, a facile one-step hydrothermal approach was established for the direct growth of hexagonal-phase WO_3_ nanostructures on FTO substrates. Through precise control of the precursor composition—specifically the ratios of the self-seeding and capping agents—the resulting WO_3_ films exhibited enhanced morphology, crystallinity, and electrochromic performance. The resulting WO_3_ films display high-performance electrochromism, characterized by 82.3% optical modulation at 720 nm and a coloration efficiency of 39.72 cm^2^ C^−1^. Moreover, to complement the high-performance WO_3_ film electrode, a composite PB/SnO_2_ film was fabricated, demonstrating enhanced electrochemical properties relative to those of the pristine PB film. The EESD shows superior electrochemical properties, including a distinct color change from transparent to deep blue and a high transmittance modulation of 81.47% at 720 nm, coupled with rapid switching behavior. The electrochemical analysis reveals that the device retains a high areal capacitance of 4.98 mF cm^−2^ under a current density of 0.05 mA cm^−2^. These outstanding characteristics endow the EESD with great potential for future applications in smart energy storage systems and portable electronic devices. However, it should be noted that the long-term operational stability of the device under harsh environmental conditions (e.g., high/low temperatures and humidity levels) remains to be further explored. 

## 4. Experimental Section

### 4.1. Materials

Analytical grade reagents such as sodium tungstate dihydrate (Na_2_WO_4_·2H_2_O, 99.5%), sorbitol (99.8%), rubidium sulfate (99.9%), lithium perchlorate (99%), and propylene carbonate (99%) were sourced from Shanghai Aladdin Biochemical Technology Co., Ltd., Shanghai, China and were used as supplied. Hydrochloric acid (37%) and hydrogen peroxide (30% in water) were also used without any purification and were obtained from Sinopharm Chemical Reagent Co., Ltd., Shanghai, China. Fluorine-doped tin oxide (FTO, sheet resistance: 15 Ω/sq) and indium tin oxide (ITO, 8 Ω/sq) -coated glass substrates were supplied by Wuhan Lattice Solar Technology Co., Ltd., Wuhan, China and subjected to sequential ultrasonic cleaning in acetone, deionized water, and ethanol prior to use.

### 4.2. Preparation of WO_3_ Film

The WO_3_ film was deposited on FTO glass using a hydrothermal technique. Previous works described the process to fabricate the precursor solution [19,22]. A total of 2.3 g of Na_2_WO_4_·2H_2_O was ultrasonically dissolved in 30 mL of deionized water, followed by the addition of 3 M HCl to form a yellow precipitate. After repeated washing and centrifugation at 8000 rpm for 10 minutes, the precipitate was mixed with 4 mL H_2_O_2_ and 46 mL deionized water and ultrasonicated to obtain a clear precursor solution. Clean FTO substrates were immersed in the solution, facing downward against the Teflon liner, and the sealed autoclave was heated at 120 °C for 150 minutes. WO_3_ films were hydrothermally grown on FTO substrates. Following a reported method, 2.3 g of Na_2_WO_4_·2H_2_O was dissolved in 30 mL of deionized water under ultrasonication. Adding 10 mL of 3 M HCl induced the formation of a yellow precipitate, which was repeatedly washed with deionized water and centrifuged at 8000 rpm for 10 minutes. The precipitate was then dispersed in 4 mL of H_2_O_2_ and 46 mL of deionized water, and ultrasonicated to yield a clear peroxopolytungstic acid solution. Clean FTO glass substrates were immersed in the solution, with the conductive side facing the Teflon liner. The autoclave was sealed and heated at 120 °C for 150 min. To optimize sorbitol content, 0, 1, 1.5, 2, and 3 g were added to the precursor solution, yielding film samples labeled WO_3_-SLC_0_, WO_3_-SLC_1_, WO_3_-SLC_1.5_, WO_3_-SLC_2_, and WO_3_-SLC_3_, respectively. Under the optimal sorbitol condition, rubidium sulfate was introduced at 0.1, 0.2, and 0.3 g to investigate its effect. The resulting films were designated WO_3_-Rb_0.1_, WO_3_-Rb_0.2_, and WO_3_-Rb_0.3_.

### 4.3. Preparation of SnO_2_ Films

A precursor solution was prepared by dissolving 0.5 g of urea in 40 mL of deionized water, followed by the sequential addition of 10 μL of thioglycolic acid (TGA) as a morphology modifier, 0.5 mL of 12 M HCl, and 0.05 g of SnCl_2_·2H_2_O. The mixture was stirred magnetically for 30 min until a clear solution was obtained [33]. Pre-cleaned FTO substrates were placed, angled with the conductive side down, in a 100 mL Teflon-lined autoclave containing the precursor. Hydrothermal treatment was performed at 120 °C for 1.5 h, and then the substrate was cooled naturally. The samples were rinsed, dried at 60 °C, and annealed at 400 °C for 3 h to form SnO_2_ nanosheets.

### 4.4. Preparation of PB/SnO_2_ Films

The fabrication process of WO_3_ films is shown in Figure S6. A precursor solution was prepared by dissolving 0.328 g of K_3_[Fe(CN)_6_], 0.37 g of KCl, and 0.162 g of FeCl_3_ in 100 mL of deionized water, followed by 10 min of magnetic stirring and 20 min of ultrasonication. The SnO_2_ nanosheet-coated FTO glass, a platinum foil, and an Ag/AgCl electrode were used as the working, counter, and reference electrodes, respectively. Prussian blue (PB) films were electrodeposited onto the SnO_2_ substrate at a current density of 25 μA cm^−2^ for 600 s to obtain the PB/SnO_2_ composite film.

### 4.5. EESD Assembly

The electrochromic energy storage device (EESD) was assembled with a WO_3_-based film as the negative electrode and a PB/SnO_2_ film as the positive electrode. A 3M tape spacer separated the electrodes, and 1 M LiClO_4_ in propylene carbonate (PC) was injected into the gap using a syringe as the electrolyte. The device was sealed using UV-curable adhesive.

### 4.6. Characterization

Surface and cross-sectional morphologies were examined by field-emission scanning electron microscopy (SEM, Zeiss Sigma 500, Carl Zeiss, Oberkochen, Germany). The optical transmittance spectra of the films (three-electrode setup) and electrochromic devices (two-electrode setup) were recorded using a UV–Vis spectrophotometer (Shimadzu UV-2700i, Shimadzu Corp., Kyoto, Japan) coupled with an electrochemical workstation (CHI760e, CH Instruments, Shanghai, China). Crystal structures were analyzed by X-ray diffraction (XRD, PANalytical X’Pert Pro MPD, Almelo, The Netherlands) using Cu Kα radiation. Long-term cycling stability was evaluated using a battery testing system (NEWARE CT-4008-5V6A-S1, Neware Technology Co., Ltd., Shenzhen, China). To evaluate the electrochemical performance of the electrode and the supercapacitor device, the areal capacitance was calculated from galvanostatic charge–discharge (GCD) curves using the following equation:

(2) $$C_{a}=\frac{I\times ∆t}{A\times ∆V}\;(mF\;{cm}^{-2})$$

*I* represents the discharge current in milliamperes (mA), Δ*t* denotes the discharge duration in seconds (s), *A* stands for the electrode or device surface area in square centimeters (cm^2^), and Δ*V* indicates the voltage window in volts (V).

## Data Availability

Data will be made available on request.

## Supplementary Materials

The following supporting information can be downloaded at: https://www.mdpi.com/article/10.3390/ma18122871/s1, Figure S1: Cross-sectional SEM images of WO_3_-SLC_x_ films with different SLC contents: (a) WO_3_-SLC_1_; (b) WO_3_-SLC_1.5_; (c) WO_3_-SLC_2_; (d) WO_3_-SLC_3_; Table S1: Cross-sectional thickness values of WO_3_-SLC_x_ films; Figure S2: XPS spectrum of WO_3_-SLC_1.5_; Figure S3: Cross-sectional SEM images of WO_3_-Rb_x_ films with different Rb_2_SO_4_ contents: (a) WO_3_-Rb_0.1_; (b) WO_3_-Rb_0.2_; (c) WO_3_-Rb_0.3_; Table S2: Cross-sectional thickness values of WO_3_-Rb_x_ films; Figure S4: Cross-sectional SEM images: (a) SnO_2_; (b) PB/SnO_2_; Table S3: Measured cross-sectional thicknesses of SnO_2_ and PB/SnO_2_ films; Figure S5: The XPS survey spectra.
