# Supplementary material for: High-Performance Electrochromic Energy Storage Devices Based on Hexagonal WO3 and SnO2/PB Composite Films
Source: Materials (Basel). 2025 Jun 17;18(12):2871. doi: 10.3390/ma18122871 (PMC12195387; doi:10.3390/ma18122871)
Supplement: Supplementary file 1 [file materials-18-02871-s001.zip › materials-3693090-supplementary.pdf]

materials

Article

High-Performance Electrochromic Energy Storage Devices Based on Hexagonal WO_3 and SnO_2/PB Composite Films

Yi Wang [1], Zilong Zhang [1], Ze Wang [1], Yujie Yan [1,*], Tong Feng [2,*] and An Xie [1]

[1] Key Laboratory of Functional Materials and Applications of Fujian Province, School of Materials Science and Engineering, Xiamen University of Technology, Xiamen 361024, PR China; yiwang@xmut.edu.cn (Y.W.); yujieyan@xmut.edu.cn (Y.Y.); anxie@xmut.edu.cn (A.X.)
[2] School of Mechanical Electrical and Information Engineering, Xiamen Institute of Technology, Xiamen, 361021, PR China; fengtong@xit.edu.cn
* Correspondence: fengtong@xit.edu.cn (T.F.); yujieyan@xmut.edu.cn (Y.Y.)

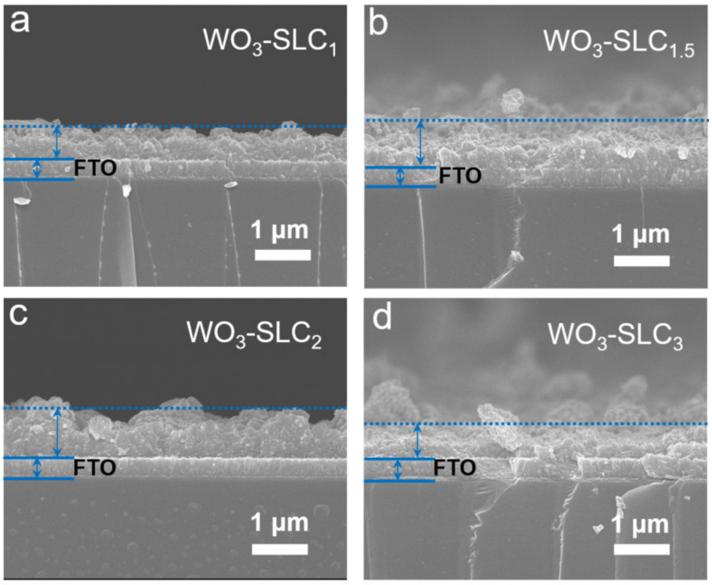

Figure S1. Cross-sectional SEM images of WO_3-SLC_x films with different SLC contents: (**a**) WO_3-SLC_1; (**b**) WO_3-$SLC_{1.5}$; (**c**) WO_3-SLC_2; (**d**) WO_3-SLC_3.

Table S1. Cross-sectional thickness values of WO_3-SLC_x films.

Sample	WO_3-SLC_1	WO_3-$SLC_{1.5}$	WO_3-SLC_2	WO_3-SLC_3
film thickness（μm）	0.67	1.01	1.10	0.62

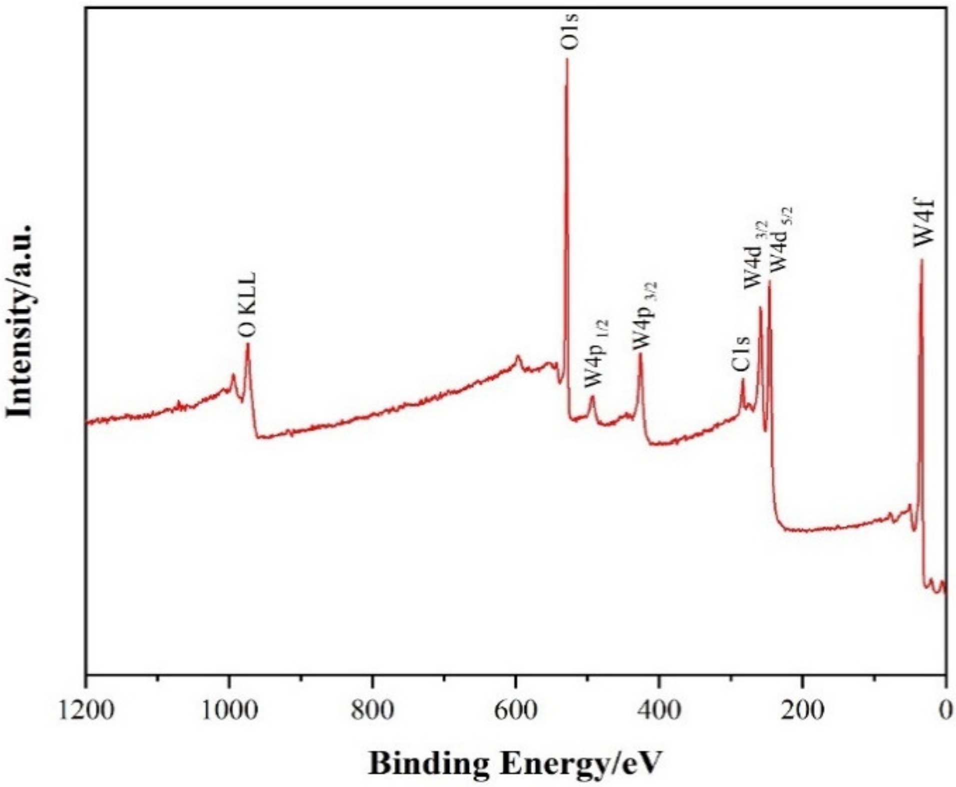

Figure S2. XPS spectrum of WO_3-$SLC_{1.5}$.

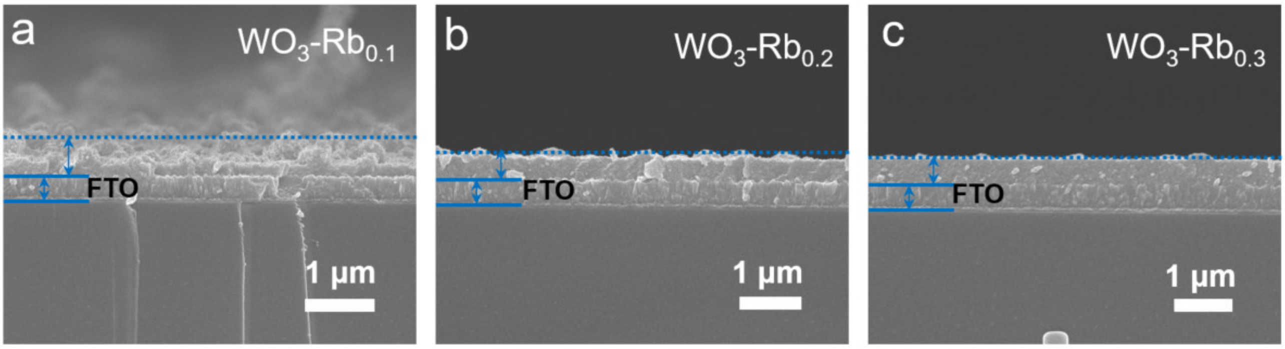

Figure S3. Cross-sectional SEM images of WO_3-Rb_x films with different Rb_2SO_4 contents: (**a**) WO_3-$Rb_{0.1}$; (**b**) WO_3-$Rb_{0.2}$; (**c**) WO_3-$Rb_{0.3}$.

Table S2. Cross-sectional thickness values of WO_3-Rb_x films.

Sample	WO_3-$Rb_{0.1}$	WO_3-$Rb_{0.2}$	WO_3-$Rb_{0.3}$
film thickness（μm）	0.65	0.50	0.46

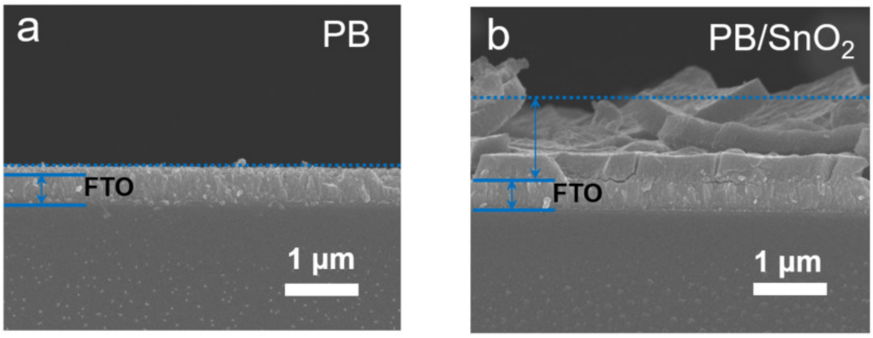

Figure S4. Cross-sectional SEM images: (**a**) SnO_2; (**b**) PB/SnO_2.

Table S3. Measured cross-sectional thicknesses of SnO_2 and PB/SnO_2 films.

Sample	SnO_2	PB/SnO_2
film thickness（μm）	0.02	1.21

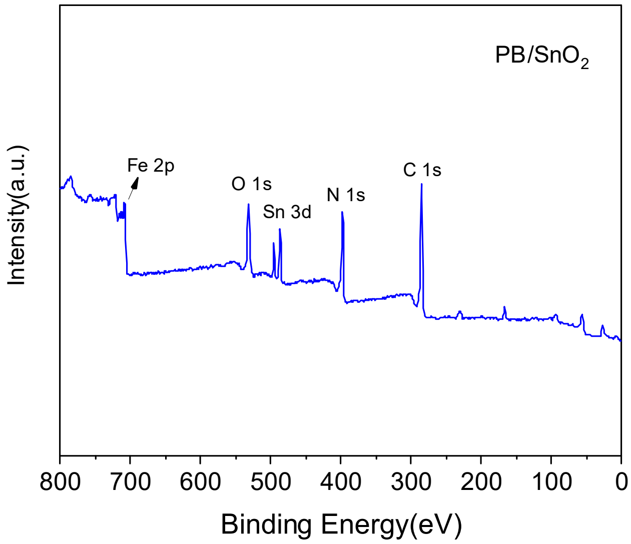

Figure S5. The XPS survey spectra.
